# Antimicrobial resistance profiles of *Staphylococcus* spp. and *Escherichia coli* isolated from dogs and cats in Seoul, South Korea during 2021–2023

**DOI:** 10.3389/fvets.2025.1563780

**Published:** 2025-08-07

**Authors:** Ye-Ram Seo, Sue-young Choi, Sori Kim, Kyoung-suk Kang, Chang-seek Ro, Ji-Yeon Hyeon

**Affiliations:** ^1^College of Veterinary Medicine, Konkuk University, Seoul, Republic of Korea; ^2^Seoul Metropolitan Government Research Institute of Public Health and Environment, Seoul, Republic of Korea

**Keywords:** antibiotic resistance, companion animal, *E. coli*, *Staphylococcus* spp., multi-drug resistance

## Abstract

We investigated the antimicrobial resistance (AMR) rates and profiles of *Staphylococcus* spp. and *Escherichia coli* (*E.coli*) isolated from dogs and cats in Seoul, South Korea during 2021–2023. We analyzed AMR profiles of *Staphylococcus* spp. and *E. coli* isolated from 2,180 samples (1,859 canine and 321 feline) collected from 36 veterinary clinics in Seoul, South Korea, as part of the Korean Veterinary Antimicrobial Resistance Monitoring System (2021–2023). A total of 484 *Staphylococcus* spp. isolates and 158 *E. coli* isolates were identified and used for AMR test. *Staphylococcus* spp. isolates exhibited the highest resistance to penicillin in both dogs (85%) and cats (29.81%). Multidrug-resistant (MDR) *Staphylococcus* spp. was more prevalent in dogs (65%) than in cats (14.42%), with three *S. pseudintermedius* isolates from dogs and a *S. pseudintermedius* isolate from a cat showing resistance to eight antibiotic classes. Methicillin-resistant *S. pseudintermedius* (MRSP) constituted 105 out of 284 *S. pseudintermedius* isolates (36.97%) in dogs and seven strains out of 14 (50%) in cats. *E. coli* isolates demonstrated the highest resistance to cefalexin in both dogs (61.72%) and cats (56.67%). The prevalence of MDR *E. coli* was higher in dogs (37.5%) than in cats (26.67%). This study highlights the concerning prevalence of AMR in commensal or potentially opportunistic pathogens from companion animals, particularly in dogs. It is crucial to promote the prudent use of antimicrobials in companion animals and ensure the ongoing monitoring of trends in antimicrobial-resistant bacteria to mitigate the selection and spread of antibiotic-resistant bacteria between humans and companion animals.

## 1 Introduction

Antimicrobial resistance (AMR) is recognized by the World Health Organization (WHO) as one of the top global public health and development threats (1, 2). AMR in animals has increased due to the overuse and misuse of antibiotics not only in food-producing animals (3–5) but also in companion animals (6). In particular, antimicrobial agents used in human medicine have been frequently used in small animal veterinary practice, with a significant reliance on broad-spectrum agents (5). This perspective has drawn increased attention to companion animals, mainly dogs and cats, as potential reservoirs of AMR due to their close contact with humans and frequent exposure to broad-spectrum antimicrobials (3, 5). In recent years, the emergence of multidrug-resistant (MDR) bacteria in dog and cat populations has posed a significant threat, contributing to therapeutic failures and prolonged hospitalization periods (7–9). In response, the World Organization for Animal Health (WOAH) revised its international standards on AMR, expanding its scope to include companion animals. These revised standards provide specific recommendations for the prudent use of critically important antimicrobial agents in veterinary medicine to protect both animal and human health^1^. Therefore, the investigation of AMR in companion animals is crucial from both veterinary and human medicine perspectives.

To monitor AMR, indicator bacteria such as *Staphylococcus* spp. and *Escherichia coli* (*E. coli*) have been used across various sources, including companion animals (1), pig farms (10), wildlife (11), raw milk (12), foodstuffs (13), patients with bacteremia (14), and hospital environments (15). *E. coli* is a commensal or potentially opportunistic pathogen commonly found in the intestinal tract of animals and humans (10). Similarly, different *Staphylococcus* spp. are part of the commensal microbiota but can also cause diseases ranging from abscesses and mastitis to septicemia (10). In recent decades, the prevalence of AMR in *Staphylococcus* spp., particularly methicillin resistance, has significantly increased in both human and veterinary patients (16).

According to a report from the Seoul Digital Foundation, 612,000 of South Korea’s 3,500,000 registered pet dogs (17.5%) reside in Seoul, and the ratio of pet dogs to households in Seoul is 14.9%, indicating that more than one in 10 households owns a pet dog.^2^ Densely populated cities like Seoul can serve as a reservoir for antibiotic-resistant bacteria, as dogs and cats live in close proximity to humans, increasing the risk of microorganism transmission (17). This study aimed to investigate the AMR rates and resistance profiles of *Staphylococcus* spp. and *E. coli* isolated from dogs and cats in Seoul, South Korea, between 2021 and 2023, and as part of the Korean Veterinary Antimicrobial Resistance Monitoring System.

## 2 Materials and methods

### 2.1 Sample collection

Clinical samples were collected from 36 veterinary clinics in Seoul participating in the Korean Veterinary Antimicrobial Resistance Monitoring System between 2021 and 2023 (15 clinics in 2021, 15 in 2022, and 20 in 2023). Samples included diarrhea, skin swabs, nasal swabs, and urine collected from dogs and cats exhibiting symptoms relevant to these sample types. In total, 1,859 samples were collected from dogs and 321 from cats (Table 1). However, cat urine samples were excluded from the monitoring system due to challenges associated with sample collection. All samples were placed on ice immediately after collection and transported to the Seoul Research Institute of Public Health and Environment within 6 h.

**Table 1:** The number of clinical samples from dogs and cats collected between 2021 and 2023 and prevalence of *Staphylococcus* spp. and *E. coli* in the samples.

Clinical samples	No. of collected samples				No. of positive samples (%)	
	2021	2022	2023	Total	*Staphylococcus* spp.	*E. coli*
Dogs						
Diarrhea	52	24	57	133	UD^†^	59 (44.36)
Skin swab	345	182	165	692	277 (40.03)	UD
Urine	245	237	276	758	43 (5.67)	69 (9.10)
Nasal swab	115	59	102	276	60 (21.74)	UD
Total	797	537	633	1,859	380 (22.02)	128 (14.37)
Cats						
Diarrhea	16	6	10	32	UD	30 (93.75)
Skin swab	29	88	73	190	53 (27.89)	UD
Nasal swab	15	45	39	99	51 (51.52)	UD
Total	80	154	122	321	104 (35.99)	30 (93.75)

^†^UD: Undetermined.

### 2.2 Bacteria isolation and identification

Skin and nasal swabs, urine samples from dogs, as well as skin and nasal swabs from cats, were collected for the isolation of *Staphylococcus* spp. Diarrheic fecal samples from both dogs and cats, along with urine from dogs, were used to isolate *E. coli*. Diarrhea samples were collected using transported fecal swabs (470 CE, Copan Italy Spa, Brescia, Italy) and plated onto eosin methylene blue agar (Thermo Fisher Scientific, Milan, Italy) for *E. coli* isolation. Other clinical samples were inoculated onto MacConkey agar and blood agar (Thermo Fisher Scientific) using sterilized cotton swabs. All culture plates were incubated aerobically at 37°C overnight. After incubation, presumptive *E. coli* and *Staphylococcus* spp. colonies were identified using matrix-assisted laser desorption/ionization-time of flight mass spectrometry (MALDI-TOF MS). For MALDI-TOF analysis, a single colony was directly applied onto the target plate, overlaid with 1 μL of matrix solution. The sample was allowed to air-dry at room temperature prior to analysis. Spectra acquisition was performed using the Bruker MALDI-TOF system (Bruker Corporation, Billerica, MA, USA) following the manufacturer’s protocol. Identification of isolates was carried out with the MALDI Biotyper MBT Compass 4.1 software. Isolates yielding a score ≥2.0 were considered reliably identified at the species level, while those with scores ranging from 1.7 to 1.99 were classified at the genus level and subjected to further confirmation using the VITEK 2 Compact system (bioMérieux, Marcy-l’Étoile, France).

### 2.3 Antibiotic susceptibility test

Isolates collected in 2021 and 2022 were tested using the Sensititre™ MIC panel system (Thermo Fisher Scientific), with COMPGP1F for *Staphylococcus* spp. and COMPGN1F for *E. coli* to determine the minimum inhibition concentrations (MICs) of the isolates. Isolates collected in 2023 were analyzed using the VITEK 2 Compact system, employing GP80 AST cards for *Staphylococcus* spp. and GN97 AST cards for *E. coli*. In this study, we report the results of antimicrobial susceptibility testing for 13 agents common to both systems: for *Staphylococcus* spp.—gentamicin, oxacillin + 2% NaCl, penicillin, cefovecin, chloramphenicol, clindamycin, enrofloxacin, marbofloxacin, pradofloxacin, nitrofurantoin, erythromycin, trimethoprim/sulfamethoxazole, and amoxicillin/clavulanic acid; and for *E. coli*—amikacin, gentamicin, ampicillin, imipenem, cefovecin, chloramphenicol, doxycycline, tetracycline, enrofloxacin, marbofloxacin, pradofloxacin, trimethoprim/sulfamethoxazole, and amoxicillin/clavulanic acid.

For the Sensititre MIC panel, bacterial suspensions were adjusted to a 0.5 McFarland standard. The inoculum (10 μL) was added to a tube containing 11 mL of Sensititre™ Cation-Adjusted Mueller-Hinton Broth with TES and thoroughly mixed, and 50 μL was dispensed into each well of a 96-well plate. The plates were sealed and incubated aerobically at 35°C for 18–24 h. The results were interpreted using the Sensititre Vizion™ Digital MIC Viewing System. All results were interpreted according to the guidelines of the Korean Animal and Plant Quarantine Agency, which are based on Clinical and Laboratory Standards Institute (CLSI) documents VET01S (18) and M100 breakpoints (19) (Supplementary Table 1).

For the VITEK 2 system, bacterial suspensions were adjusted to a 0.6 McFarland standard. For gram-negative isolates, 145 μL of the suspension was added to 3 mL of sterile saline; for gram-positive isolates, 280 μL was used. The inoculum of *Staphylococcus* spp. was loaded onto a GP80 AST card, and that of *E. coli* was loaded onto a GN97 AST card. AMR results were interpreted based on the “Result – Expertised” data provided by the system’s Advanced Expert System.

Intermediate susceptibility results were not classified as resistant. In prevalence analyses, only isolates explicitly categorized as “resistant” were considered resistant. Intermediate results were grouped with susceptible isolates unless otherwise stated. MDR was defined as resistance to at least one antimicrobial agent in three or more different antimicrobial classes, excluding intrinsic resistance, per the criteria proposed by Magiorakos et al. (20).

Quality control was performed for the Sensititre™ MIC panel system using the following ATCC reference strains following the manufacturer’s instructions: *S. aureus* ATCC 29213, *Enterococcus faecalis* (*E. faecalis*) ATCC 29212, *E. coli* ATCC 25922, *E. coli* ATCC 35218, *Pseudomonas aeruginosa* ATCC 27853, and *Klebsiella pneumoniae* ATCC 700603. For the VITEK 2 Compact system, *E. faecalis* ATCC 29212 and *E. coli* ATCC 25922 were used. All quality control results were within CLSI-defined acceptable ranges throughout the study period.

### 2.4 *mecA* polymerase chain reaction (PCR)

To distinguish methicillin-resistant *Staphylococcus pseudintermedius* (*S. pseudintermedius*, MRSP), the presence of the *mecA* gene was investigated in all oxacillin-resistant *S. pseudintermedius* isolates using PCR, following a modified protocol by Lee et al. (21).

For direct colony PCR, individual colonies grown on blood agar plates were used directly as templates, and sterilized water was included as a negative control. Each PCR reaction was performed in a final volume of 50 μL, containing 0.2 μM of *mecA*-specific primers (mecA-MR1: 5′-ATGAGATTAGGCATCGTTCC-3′ and mecA-MR2: 5′-TGGATGACAGTACCTGAGCC-3′) (21) and 25 μL of 2 × EmeraldAmp® GT PCR Master Mix (Takara, Otsu, Japan). The PCR conditions were as follows: initial denaturation at 94°C for 5 min; followed by 30 cycles of denaturation at 94°C for 30 s, annealing at 55°C for 30 s, and extension at 72°C for 30 s; and a final extension at 72°C for 7 min. PCR products were analyzed by electrophoresis on 2% agarose gels containing SafeView™ Classic stain (ABM, Richmond, BC, Canada). Samples showing a positive band at 554 bp were subjected to gel extraction using the GeneJET Gel Extraction Kit (Thermo Fisher Scientific), and the identity of the *mecA* gene was confirmed by sequencing.

### 2.5 Statistical methods

Statistical analysis was performed using MedCalc® Statistical Software version 23.0.6. Differences in antibiotic resistance rates between clinical sample types from dogs and cats were evaluated using a two-way chi-square test, with *p*-values < 0.05 considered significant.

## 3 Results

### 3.1 Prevalence of *Staphylococcus* spp. in the samples

The prevalence and species distribution of *Staphylococcus* spp. isolated from clinical samples collected from dogs and cats are summarized in Tables 1, 2. *Staphylococcus* spp. were identified in 380 out of 1,726 samples (22.02%) obtained from dogs, which included 692 skin swabs, 758 urine samples, and 276 nasal swabs. In cats, *Staphylococcus* spp. were detected in 104 out of 289 samples (35.99%), consisting of 190 skin swabs and 99 urine samples. Among canine samples, the highest isolation rates were observed in skin swabs (40.03%), followed by nasal swabs (21.74%) and urine samples (5.67%). In cats, nasal swabs exhibited the higher isolation rate (51.52%) than skin swabs (27.89%) (Table 1).

**Table 2:** Antibiotic resistance of *Staphylococcus* spp. isolated from dogs and cats in this study (*n* = 484).

Antibiotics	Dogs								Cats					
	Urine (*n* = 43)		Skin swabs (*n* = 277)		Nasal swabs (*n* = 60)		Total (*n =* 380)		Skin swabs (*n* = 53)		Nasal swabs (*n* = 51)		Total (*n =* 104)	
	No.	%	No.	%	No.	%	No.	%	No.	%	No.	%	No.	%
Non-MDR	8	18.60	101	36.46	24	40	133	35	46	84.31	43	84.31	89	85.58
MDR	35	81.40	176**	63.54	36**	60	247**	65	7	15.69	8	15.69	15	14.42
Gentamicin	9	20.93	63*	22.76	12	20	84**	22.11	4	7.55	0	0	4	3.85
Oxacillin	19	44.19	94*	33.94	23**	38.34	136**	35.79	10	18.87	8	15.69	18	17.31
Penicillin	41	95.35	233**	84.12	49**	81.67	323**	85	17	32.08	14	27.45	31	29.81
Cefovecin	19	44.19	91*	32.85	21*	35	131**	34.47	9	16.98	9	17.65	18	17.31
Chloramphenicol	25	58.14	117**	42.24	25**	41.67	167**	43.95	6	11.32	5	9.80	11	10.58
Clindamycin	29	67.44	133**	48.01	30**	50	192**	50.53	6	11.32	6	11.76	12	11.54
Enrofloxacin	19	44.19	86**	31.05	21**	35	126**	33.16	5	9.43	5	9.80	10	9.62
Marbofloxacin	20	67.44	88**	31.77	22**	36.67	130**	34.21	5	9.43	4	7.84	9	8.65
Pradofloxacin	14	32.56	64**	23.1	18**	30	96**	25.26	2	3.77	4	7.84	6	5.77
Nitrofurantoin	0	0	4	1.44	1	1.67	5	1.32	2	3.77	0	0	2	1.92
Erythromycin	31	72.09	155**	55.96	35**	58.33	221**	58.16	5	9.43	7	13.73	12	11.54
Trimethoprim/Sulfamethoxazole	30	69.77	144**	51.99	25**	41.67	199**	52.37	3	5.66	3	5.88	6	5.77
Amoxicillin/clavulanic acid	13	30.23	62	22.38	15	25	90*	23.68	6	11.32	7	13.73	13	12.5

Statistical differences of antibiotic resistance rates between dogs and cats. *p-value < 0.05, **p-value < 0.01.

*S. pseudintermedius* was the most prevalent *Staphylococcus* species isolated from dogs, accounting for 284 out of 380 isolates (74.74%) across all sample types (Supplementary Table 2). *S. schleiferi* was the second most frequently isolated species in urine and skin swab samples, while *S. felis* was the second most common in nasal swabs. In cats, *S. felis* was predominant, comprising 61 out of 104 isolates (58.65%) across all sample types. *S. pseudintermedius* was the second most prevalent species in skin swabs and *S. aureus* was the second most frequently detected species in nasal swabs (Supplementary Table 2). The distribution of *Staphylococcus* species varied by sample type, with nasal swabs showing the highest species diversity in both dogs and cats (Supplementary Table 2).

### 3.2 Antibiotic resistance of *Staphylococcus* spp.

The antibiotic resistance rates and the MIC distribution of *Staphylococcus* spp. in dogs and cats are summarized in Table 2 and Supplementary Table 3. Overall, 247 out of 380 isolates (65%) from dogs and 15 out of 104 isolates (14.42%) from cats were resistant to at least one agent in three or more antimicrobial categories, classifying them as MDR (Table 2). Statistical analysis revealed significantly higher overall MDR rates as well as MDR rates by sample type, in dogs than in cats (*p* < 0.00001). Penicillin exhibited the highest resistance rate across all sample types in isolates from both dogs and cats, while nitrofurantoin showed the lowest resistance rate (Table 2). In dogs, isolates exhibited the highest resistance to penicillin (85%), followed by erythromycin (58.16%), trimethoprim/sulfamethoxazole (52.37%), and clindamycin (50.53%). In cats, resistance was most frequently observed to penicillin (29.81%), followed by oxacillin and cefovecin (17.31% each). Comparative analysis of *Staphylococcus* spp. isolates from the same clinical sample types in dogs and cats revealed that resistance rates to most tested antibiotics were significantly higher in isolates from dogs than from cats, with the exception of nitrofurantoin (*p* < 0.05) (Table 2).

Oxacillin-resistant *Staphylococcus* spp. isolated from various clinical samples in dogs and cats are summarized in Table 3. For *S. pseudintermedius*, the most commonly isolated species from dog, oxacillin resistance was observed in 73 out of 211 isolates (34.60%) from skin swabs, 15 out of 34 isolates (44.12%) from nasal swabs, and 17 out of 39 isolates (43.59%) from urine samples. In cats, oxacillin resistance among *S. pseudintermedius* was recorded in 5 out of 9 isolates (55.56%) from skin swabs and 2 out of 5 isolates (40%) from nasal swabs. Oxacillin-resistant *S. pseudintermedius* harboring *mecA* gene was 101 out of 284 *S. pseudintermedius* isolates (35.56%) in dogs and 7 out of 14 (50%) in cats (data not shown).

**Table 3:** Oxacillin-resistant *Staphylococcus* spp. isolated from dogs and cats in this study.

Species	No. of oxacillin-resistant *Staphylococcus* spp./No. of total *Staphylococcus* spp. isolated in this study (%)						
	Dogs				Cats		
	Urine (*n* = 43)	Skin swabs (*n* = 277)	Nasal swabs (*n* = 60)	Total (*n* = 380)	Skin swabs (*n* = 53)	Nasal swabs (*n* = 51)	Total (*n* = 104)
*S. aureus*	–	–	0/3 (0)	0/3 (0)	–	3/8 (37.5)	3/8 (37.5)
*S. capitis*	–	–	2/2 (100)	2/2 (100)	1/1 (100)	1/2 (50)	1/3 (33.34)
*S. caprae*	–	0/1 (0)	–	0/1 (0)	–	–	
*S. cohnii*	–	1/1 (100)	1/1 (100)	2/2 (100)	–	–	
*S. delphini*	–	–	–	–	–	0/1 (0)	0/1 (0)
*S. epidermidis*	–	–	1/1 (100)	1/1 (100)	0/1 (0)	–	0/1 (0)
*S. equorum*				–	1/1 (100)		0/1 (0)
*S. felis*	–	0/2 (0)	1/10 (8.33)	1/12 (8.34)	1/34 (2.94)	0/27 (0)	1/61 (1.64)
*S. haemolyticus*	–	–	2/2 (100)	2/2 (100)	–	–	
*S. hominis*	–	–	–	–	–	0/2 (0)	0/2 (0)
*S. pseudintermedius*	17/39 (43.59)	73/211 (34.60)	15/34 (44.12)	105/284 (36.97)	5/9 (55.56)	2/5 (40)	7/14 (50)
*S. saprophyticus*	–	–	–	–	–	1/1 (100)	0/1 (0)
*S. schleiferi*	2/4 (50)	20/62 (32.26)	1/4 (25)	23/70 (32.86)	2/6 (33.33)	1/1 (100)	3/7 (42.86)
*S. sciuri*	–	–	0/1 (0)	0/1 (0)	–	–	
*S. simulans*	–	–	–		–	0/3 (0)	0/3 (0)
*S. warneri*	–	–	0/2 (0)	0/2 (0)	–	–	
*S. xylosus*	–	–	–		0/1 (0)	0/1 (0)	0/2 (0)
Total	19/43 (44.19)	94/277 (33.94)	23/60 (38.33)	136/380 (35.79)	10/53 (18.87)	8/51 (15.69)	18/104 (17.31)

The antibiotic resistance patterns of *Staphylococcus* spp. are summarized in Supplementary Table 4. Among the isolates, 37 out of 380 isolates (9.74%) from dogs and 65 out of 104 isolates (62.5%) from cats were susceptible to all tested antibiotics. The highest proportions of resistant strains were observed for β-lactams in dogs (48 out of 380, 12.11%) and in cats (12 out of 104, 11.54%) (Supplementary Table 4). The most prevalent MDR pattern in dogs was β-lactams-amphenicols-lincosamides-macrolides-trimethoprim/sulfamethoxazole, identified in 32 out of 247 MDR isolates (12.96%). Meanwhile in cats, the most common pattern was β-lactams-quinolones-amoxicillin/clavulanic acid, observed in 3 out of 15 MDR isolates (20%) (Supplementary Table 4). Notably, three *S. pseudintermedius* isolates from dogs and a *S. pseudintermedius* isolate from a cat exhibited resistance to 8 antibiotic classes (Supplementary Table 4).

### 3.3 Prevalence and antibiotic resistance of *Escherichia coli*

The prevalence of *E. coli* in dogs and cats is summarized in Table 1. In dogs, *E. coli* was isolated from 128 out of 891 total samples (14.37%), which included 59 out of 133 diarrhea samples (44.36%) and 69 out of 758 urine samples (9.10%). In cats, *E. coli* was isolated from 30 out of 32 diarrhea samples (93.75%).

The antibiotic resistance rates and the MIC distribution of *E. coli* from dogs and cats are presented in Table 4 and Supplementary Table 5, respectively. Overall, 48 out of 128 isolates (37.5%) from dogs and 8 out of 30 isolates (26.67%) from cats were classified as MDR. The MDR rate in urine isolates (30 out of 69, 43.48%) was higher than that in diarrhea isolates (18 out of 59, 30.51%). *E. coli* isolates from diarrhea samples exhibited the highest resistance rates to cefalexin, with 50 out of 59 isolates (84.75%) from dogs and 17 out of 30 isolates (56.67%) from cats, showing a significant difference between the isolates from the two host species (*p* <0.01) (Table 4). No resistance to amikacin or imipenem was detected across all host species and sample types (Table 4). In dogs, *E. coli* isolates from diarrhea samples showed no resistance to ampicillin or amoxicillin/clavulanic acid. However, urine isolates exhibited the highest resistance to ampicillin, with 43 out of 69 isolates (62.32%) displaying resistance (Table 4). In cats, high resistance rates were observed against cefovecin and cefpodoxime, with 11 out of 30 isolates (36.67%) being resistant to each. These results suggest a high antibiotic resistance to cephalosporins in isolates from cats (Table 4).

**Table 4:** Antibiotic resistance of *E. coli* isolated from dogs and cats in this study (*n* = 158).

Antibiotics	Dogs						Cats	
	Urine (*n* = 69)		Diarrhea (*n* = 59)		Total (*n* = 128)		Diarrhea (*n* = 30)	
	No.	%	No.	%	No.	%	No.	%
Non-MDR	39	56.52	41	69.49	80	62.5	22	73.33
MDR	30	43.38	18	30.51	48	37.5	8	26.67
Amikacin	0	0	0	0	0	0	0	0
Gentamicin	13	18.84	10	16.95	23	17.97	4	13.33
Ampicillin	43	62.32	0	0	43	33.59	4	13.33
Imipenem	0	0	0	0	0	0	0	0
Cefovecin	29	42.03	12	20.34	41	32.03	11	36.67
Cefpodoxime	30	43.48	12	20.34	42	32.81	11	36.67
Cefalexin	31	44.93	50*	84.75	81	61.72	17	56.67
Chloramphenicol	8	11.59	10	16.95	18	14.06	4	13.33
Doxycycline	13	18.84	11	18.64	24	18.75	3	10
Tetracycline	18	26.09	19	32.20	37	28.91	8	26.67
Enrofloxacin	30	43.48	14	23.73	44	34.38	8	26.67
Marbofloxacin	28	40.58	14	23.73	42	32.81	5	16.67
Pradofloxacin	28	40.58	14	23.73	42	32.81	6	20
Trimethoprim/Sulfamethoxazole	17	24.64	14	23.73	31	24.22	6	20
Amoxicillin-clavulanic acid	20	28.99	0	0	20	15.63	2	6.67

Statistical differences of antibiotic resistance rates between dogs and cats: *p*-value < 0.05, *.

The antibiotic resistance pattern of *E. coli* isolates revealed that 25 out of 128 isolates (19.531%) from dogs and 10 out of 30 isolates (33.33%) from cats were susceptible to all tested antibiotics (Supplementary Table 6). The most common resistance pattern was exclusively against β-lactams, observed in 27 out of 128 isolates (21.10%) from dogs and 6 out of 30 isolates (20.0%) from cats (Supplementary Table 6). Six isolates from dogs and two isolates from cats exhibited resistance to 6 antibiotic classes (Supplementary Table 6).

## 4 Discussion

Seoul is the capital city of South Korea, a densely populated metropolis with a population of 9.619 million people (in 2024) living in a land area of 605.21 km^2^.^3^ According to the Korea Rural Economic Institute, the proportion of the total population that owns pets has increased from 17.4% in 2010 to 27.7% in 2020. As both human and pet populations continue to grow in Seoul, monitoring trends in antimicrobial-resistant bacteria in companion animals is increasingly important for public and animal health. In this study, we investigated the prevalence and AMR patterns of *Staphylococcus* spp. and *E. coli* isolated from clinical samples of dogs and cats collected from veterinary clinics in Seoul, South Korea, between 2021 and 2023 as part of the Korean Veterinary Antimicrobial Resistance Monitoring System. Notably, due to the transition in antimicrobial susceptibility testing methodology within the Korean Veterinary Antimicrobial Resistance Monitoring System in 2023, different antimicrobial susceptibility testing methods were used for isolates collected in different years: the Sensititre™ MIC system for isolates from 2021 to 2022 and VITEK® 2 Compact system for those from 2023. Each system employs its own panel of antimicrobial concentrations and interpretive criteria. Results from the Sensititre™ system were interpreted using CLSI guidelines, while those from the VITEK® 2 system were interpreted using the Advanced Expert System. These methodological differences may affect the comparability of MIC values and resistance patterns across years and should be considered when interpreting the findings.

*Staphylococcus* spp. are commensal bacteria and opportunistic pathogens capable of zoonotic transmission (22, 23). Several studies have examined the prevalence of *Staphylococcus* spp. in both healthy and sick humans and animals (1, 24–26). Prevalence rates vary depending on the sample type and host species: ranging from 67.3 to 73.8% in healthy dogs and cats (26), to 69% in sick dogs and cats (1), and to 82.81, 76.4, and 91% in healthy domestic cats, feral cats, and sick cats, respectively (25). In the present study, the prevalence of *Staphylococcus* spp. ranged from 5.67% in canine urine samples to 51.52% in feline nasal swab samples. The predominant species identified were *S. pseudintermedius* in dogs and *S. felis* in cats, which is consistent with previous studies (14, 25). In this study, *Staphylococcus* spp. and *E. coli* isolates from dogs generally exhibited higher resistance rates than those from cats. This finding aligns with the results of a previous study by Yang et al. (27), which reported a greater abundance of antibiotic-resistance genes in canine feces than in feline feces. Notably, this study identified a high prevalence of MDR in *Staphylococcus* spp. isolated from dogs. *S. pseudintermedius* poses a potential public health concern due to its ability to transfer antibiotic resistance genes to human staphylococcal species (28, 29).

According to the “2023 Use of Antimicrobials in Domestic Veterinary Clinics” survey conducted by the Animal and Plant Quarantine Agency,^4^ the top four antibiotics with the highest sales volumes for companion animal treatment were enrofloxacin, cefalexin, ampicillin, and penicillin G. In our study, *Staphylococcus* spp. and *E. coli* isolates from dogs and cats exhibited the highest resistance rates to penicillin and cefalexin, respectively. This finding is consistent with previous studies on *E. coli* strains from fecal samples of healthy dogs and cats (30) and *Staphylococcus* spp. strains from dogs (31, 32) in South Korea. We suggest that the frequent use of these antibiotics in companion animals has significantly contributed to the selection and maintenance of resistant bacteria in South Korea. However, comprehensive clinical metadata, including prior antimicrobial treatment history of the sampled animals, were unavailable, and uniform inclusion and exclusion criteria were not applied due to the retrospective nature of sample collection from multiple clinics. As prior antibiotic exposure is known to significantly influence the prevalence of antimicrobial resistance, the absence of this information may have limited the interpretation of resistance patterns across host species or sample types.

The use of veterinary antibiotics can promote the selection and transmission of MDR to other organisms, underscoring the needs for strategies that reduce resistance selection and preserve the clinical efficacy of antibiotics. Empirical first-line treatments for urinary or skin infections in companion animals commonly include amoxicillin/clavulanate, trimethoprim/sulfadiazine, and clindamycin. Although antimicrobials are generally not indicated in most cases of diarrhea in dogs, as they are often self-limiting and resolve with supportive fluid therapy alone, broad-spectrum antibiotics such as amoxicillin/clavulanate, cefalexin, and ampicillin are still commonly prescribed in primary veterinary practice for gastrointestinal infections presenting with diarrhea (33).

In this study, *Staphylococcus* spp. exhibited high resistance to trimethoprim/sulfadiazine (52.37%) and clindamycin (50.53%), suggesting limited effectiveness of these agents for empirical treatment of opportunistic *Staphylococcus* infections in companion animals. Additionally, amoxicillin/clavulanate and ampicillin may exert a greater selective pressure on intestinal *E. coli* populations than cefalexin, potentially influencing the gut microbiota and resistance dynamics. The antimicrobial resistance data generated in this study offer valuable insights to inform more judicious selection of antibiotics for the treatment of infectious diseases in companion animals. In addition, the current study did not assess inducible clindamycin resistance in *Staphylococcus* spp. Therefore, without confirmatory testing such as the D-zone test, isolates with inducible resistance may be incorrectly classified as clindamycin-susceptible, potentially resulting in inappropriate treatment. To improve the accuracy of antimicrobial susceptibility testing, future studies should incorporate methods to detect inducible clindamycin resistance.

Although this study reports phenotypic resistance profiles for *E. coli* and *Staphylococcus* spp., molecular mechanisms of resistance in *E. coli*, such as extended-spectrum β-lactamases (ESBLs), carbapenemases, and other resistance genes, were not investigated. In *Staphylococcus* spp., resistance gene detection was limited to *mecA*. Comprehensive molecular analyses using whole-genome sequencing or targeted PCR would enhance understanding of resistance mechanisms, facilitate epidemiological tracking, and support more effective antimicrobial resistance control.

Guided by the One Health concept, which connects human, animal, and environmental health, the findings of this study offer valuable insights into the epidemiology of AMR in companion animals. The results of this study emphasize the need for integrated approaches to address this shared public health challenge. The antibiotic resistance findings in bacteria isolated from companion animals are particularly significant, as *Staphylococcus* and *E. coli* are zoonotic pathogens capable of exchanging antibiotic-resistance genes with the human microbiome (24, 34). Addressing this issue requires the implementation of robust antibiotic resistance monitoring and gene analysis in both human and companion animal populations. Our study highlights the concerning prevalence of AMR in commensal or potentially opportunistic pathogens from companion animals, particularly in dogs, where high rates of MDR were observed in Seoul, South Korea. These findings underscore the need for long-term surveillance of resistance patterns in veterinary settings to prevent the spread and evolution of antibiotic-resistant bacteria among both humans and companion animals.

## Data Availability

The raw data supporting the conclusions of this article will be made available by the authors without undue reservation.

## Footnotes

^1^ https://www.woah.org/en/revised-standards-strengthened-actions-to-contain-amr/

^2^ https://smart.seoul.go.kr/board/41/22821/board_view.do

^3^ https://english.seoul.go.kr/seoul-views/meaning-of-seoul/4-population/

^4^ https://ebook.qia.go.kr/20240930_134736
