# Supplementary material for: Antimicrobial resistance profiles of Staphylococcus spp. and Escherichia coli isolated from dogs and cats in Seoul, South Korea during 2021–2023
Source: Front Vet Sci. 2025 Aug 7;12:1563780. doi: 10.3389/fvets.2025.1563780 (PMC12367511; doi:10.3389/fvets.2025.1563780)
Supplement: Supplementary file 1 [file Table_1.docx]

Supplementary table 1. CLSI VET01S and M100-based susceptibility breakpoints for antimicrobial agents used in the Sensititre MIC Panel in this study.

| Antibiotics | *Staphylococcus* spp. | |  | *Escherichia coli* | |
| --- | --- | --- | --- | --- | --- |
|  | Breakpoints(㎍/㎖) | References |  | Breakpoints  (㎍/㎖) | References |
| Amikacin | - |  |  | ≥64 | M100 |
| Amoxicillin/clavulanic acid | ≥1/0.5 | VET01S |  | Diarrhea ≥32/16, Urine ≥16/8 | VET01S |
| Ampicillin | - | - |  | Diarrhea ≥32, Urine ≥16 | VET01S |
| Cefalexin | - | - |  | Diarrhea ≥8, Urine ≥32 | VET01S |
| Cefovecin | ≥2 | VET01S |  | ≥8 | VET01S |
| Cefpodoxime | - | - |  | ≥8 | VET01S |
| Chloramphenicol | ≥32 | VET01S |  | ≥32 | VET01S |
| Clindamycin | ≥4 | VET01S |  | - | - |
| Doxycycline | - | - |  | ≥16 | VET01S |
| Enrofloxacin | ≥4 | VET01S |  | ≥4 | VET01S |
| Erythromycin | ≥8 | M100 |  | - | - |
| Gentamicin | ≥16 | M100 |  | ≥16 | M100 |
| Imipenem | - | - |  | ≥4 | M100 |
| Marbofloxacin | ≥4 | VET01S |  | ≥4 | VET01S |
| Nitrofurantoin | ≥128 | M100 |  | - | - |
| Oxacillin | ≥0.5 | VET01S |  | - | - |
| Penicillin | ≥0.25 | VET01S |  | - | - |
| Pradofloxacin | ≥2 | VET01S |  | ≥2 | VET01S |
| Tetracycline | - | - |  | ≥16 | M100 |
| Trimethoprim/Sulfamethoxazole | ≥4/76 | M100 |  | ≥4/76 | M100 |
