# Supplementary material for: Antimicrobial resistance profiles of Staphylococcus spp. and Escherichia coli isolated from dogs and cats in Seoul, South Korea during 2021–2023
Source: Front Vet Sci. 2025 Aug 7;12:1563780. doi: 10.3389/fvets.2025.1563780 (PMC12367511; doi:10.3389/fvets.2025.1563780)
Supplement: Supplementary file 2 [file Table_2.docx]

Supplementary table 2. *Staphylococcus* spp. isolated from clinical samples from dogs and cats between 2021 and 2023 in this study (*n*=484).

| Species | Number of *Staphylococcus* spp. positive samples (%) | | | | | | | |
| --- | --- | --- | --- | --- | --- | --- | --- | --- |
|  | Dogs | | | |  | Cats | | |
|  | Urine  (*n*=43) | Skin swabs  (*n*=277) | Nasal  swabs  (*n*=60) | Total  (*n*=380) |  | Skin swabs  (*n*=53) | Nasal  swabs  (*n*=51) | Total  (*n*=104) |
| *S. aureus* | - | - | 3 (5.0) | 3 (0.79) |  | - | 8 (15.69) | 8 (7.96) |
| *S. capitis* | - | - | 2 (3.33) | 2 (0.53) |  | 1 (1.89) | 2 (3.92) | 3 (2.88) |
| *S. caprae* | - | 1 (0.36) | - | 1 (0.26) |  | - | - | - |
| *S. cohnii* | - | 1 (0.36) | 1 (1.67) | 2 (0.53) |  | - | - | - |
| *S. delphini* | - | - | - | - |  | - | 1 (1.96) | 1 (0.96) |
| *S. epidermidis* | - | - | 1 (1.67) | 1 (0.26) |  | 1 (1.89) | - | 1 (0.96) |
| *S. felis* | - | 2 (0.72) | 10 (16.67) | 12 (3.16) |  | 34 (64.15) | 27 (52.94) | 61 (58.65) |
| *S. haemolyticus* | - | - | 2 (3.33) | 2 (0.53) |  | - | - | - |
| *S. hominis* | - | - | - | - |  | - | 2 (3.92) | 2 (1.92) |
| *S. pseudintermedius* | 39 (90.7) | 211 (76.17) | 34 (56.67) | 284 (74.74) |  | 9 (16.98) | 5 (9.80) | 14 (13.46) |
| *S. saprophyticus* | - | - | - | - |  | - | 1 (1.96) | 1 (0.96) |
| *S. schleiferi* | 4 (9.3) | 62 (22.38) | 4 (6.67) | 70 (18.42) |  | 6 (11.32) | 1 (1.96) | 7 (6.73) |
| *S. sciuri* | - | - | 1 (1.67) | 1 (0.26) |  | - | - | - |
| *S. simulans* | - | - | - | - |  | - | 3 (5.88) | 3 (1.92) |
| *S. warneri* | - | - | 2 (3.33) | 2 (0.53) |  | - | - | - |
| *S. xylosus* | - | - | - | - |  | 1 (1.89) | 1 (1.96) | 2 (1.92) |
