# Supplementary material for: Antimicrobial resistance profiles of Staphylococcus spp. and Escherichia coli isolated from dogs and cats in Seoul, South Korea during 2021–2023
Source: Front Vet Sci. 2025 Aug 7;12:1563780. doi: 10.3389/fvets.2025.1563780 (PMC12367511; doi:10.3389/fvets.2025.1563780)
Supplement: Supplementary file 3 [file Table_3.pdf]

Supplementary Table 3. Distribution of MICs (μg/mL) in *Staphylococcus* spp. tested in this study (n=484).

Host	Antibiotics	MIC (μg/mℓ)*													Total
		≤0.03	0.06	0.125	0.25	0.5	1	2	4	8	16	32	64	≥128	
Dog	Gentamicin					52	3	4	154	83	58	26			380
	Oxacillin				236	18	21	20	85						380
	Penicillin	23	32	12	23	137	24	16	23	19	71				380
	Cefovecin		1	60	80	92	24	33	21	33	36				380
	Chloramphenicol								69	135	9	16	151		380
	Clindamycin			13	69	103	4	1	56	134					380
	Enrofloxacin				57	92	82	23	45	81					380
	Marbofloxacin					56	187	7	52	78					380
	Pradofloxacin			90	161	8	25	52	44						380
	Nitrofurantoin										372	3		5	380
	Erythromycin				124	28	5			223					380
	Trimethoprim/Sulphamethoxazole							103	41	79	157				380
	Amoxicillin/Clavulanic Acid				161	28	19	146	7	7	11	1			380
Cat	Gentamicin					36		1	59	4	1	3			104
	Oxacillin				85	3	2	5	9						104
	Penicillin	25	43	6	1	12	1	1	3	1	11				104
	Cefovecin		1		44	33	4	10	8	1	3				104
	Chloramphenicol								32	57	4	2	9		104
	Clindamycin			6	22	64	1		3	8					104
	Enrofloxacin				46	44	4		1	9					104
	Marbofloxacin					34	60	1	1	8					104
	Pradofloxacin			39	56	1	2	5	1						104
	Nitrofurantoin										102		1	1	104
	Erythromycin				46	40	3		1	14					104
	Trimethoprim/ Sulphamethoxazole							60		38	6				104
	Amoxicillin/Clavulanic Acid				52		4	42	2	1	3				104

*White field indicates MIC ranges tested by either the Sensititre MIC system or the VITEK® 2 Compact system. Gray areas represent concentrations not tested by either method. MIC results for isolates collected in 2021 and 2022 were interpreted according to the guidelines of the Korean Animal and Plant Quarantine Agency, based on Clinical and Laboratory Standards Institute (CLSI) documents VET01S and M100 breakpoints. For isolates collected in 2023, antimicrobial susceptibility testing was performed using the VITEK® 2 Compact system, and interpretations were based on the "Result – Expertised" output generated by the system's Advanced Expert System. For the combinations, Trimethoprim/Sulphamethoxazole and Amoxicillin/Clavulanic Acid, the concentrations indicated are those of trimethoprim and Amoxicillin, respectively.
