# Supplementary material for: Antimicrobial resistance profiles of Staphylococcus spp. and Escherichia coli isolated from dogs and cats in Seoul, South Korea during 2021–2023
Source: Front Vet Sci. 2025 Aug 7;12:1563780. doi: 10.3389/fvets.2025.1563780 (PMC12367511; doi:10.3389/fvets.2025.1563780)
Supplement: Supplementary file 4 [file Table_4.pdf]

Supplementary table 4. Multi-drug resistance of *Staphylococcus* spp. isolated from dogs and cats in this study (*n* =484).

No. of antibiotics	Antibiotic resistance patterns								Dogs				Cats			Total
									Urine	Skin swabs	Nasal swabs	Total	Skin swabs	Nasal swabs	Total	
0	None								2	26	9	37	32	33	65	102
1	Trimethoprim/Sulfamethoxazole									5		5				5
1	Quinolones									1		1	1		1	2
1	Lincosamides									2		2		1	1	3
1	β_lactams								4	37	7	48	8	4	12	60
2	Lincosamides	Macrolides									1	1				1
2	Lincosamides	Quinolones								1		1				1
2	Amphenicols	Macrolides												1	1	1
2	β_lactams	Amoxicillin/clavulanic acid								4	2	6		3	3	9
2	β_lactams	Trimethoprim/Sulfamethoxazole								6	1	7				7
2	β_lactams	Quinolones							2	12	1	15	2		2	17
2	β_lactams	Lincosamides									2	2	1		1	3
2	β_lactams	Amphenicols											2	1	3	3
2	Aminoglycosides	β_lactams								7	1	8				8
3	Lincosamides	Macrolides	Trimethoprim/Sulfamethoxazole							1		1				1
3	Amphenicols	Macrolides	Trimethoprim/Sulfamethoxazole							1		1				1
3	Amphenicols	Lincosamides	Macrolides										1		1	1
3	β_lactams	Trimethoprim/Sulfamethoxazole	Amoxicillin/clavulanic acid						1	4		5				5
3	β_lactams	Macrolides	Trimethoprim/Sulfamethoxazole							1		1				1
3	β_lactams	Quinolones	Amoxicillin/clavulanic acid							8		8	1	2	3	11
3	β_lactams	Quinolones	Trimethoprim/Sulfamethoxazole							1	1	2				2
3	β_lactams	Lincosamides	Macrolides							1	2	3		2	2	5
3	β_lactams	Amphenicols	Macrolides							1		1				1
3	Aminoglycosides	β_lactams	Amoxicillin/clavulanic acid						1	2		3				3
3	Aminoglycosides	β_lactams	Trimethoprim/Sulfamethoxazole								1	1				1
3	Aminoglycosides	β_lactams	Amphenicols							1		1				1
4	Lincosamides	Quinolones	Macrolides	Trimethoprim/Sulfamethoxazole										1	1	1
4	Amphenicols	Lincosamides	Macrolides	Trimethoprim/Sulfamethoxazole						2		2				2
4	Amphenicols	Lincosamides	Quinolones	Macrolides							1	1				1
4	β_lactams	Lincosamides	Macrolides	Amoxicillin/clavulanic acid					1		1	2				2
4	β_lactams	Lincosamides	Macrolides	Trimethoprim/Sulfamethoxazole						2		2				2
4	β_lactams	Lincosamides	Quinolones	Amoxicillin/clavulanic acid						2		2				2
4	β_lactams	Lincosamides	Quinolones	Macrolides						1		1				1
4	β_lactams	Amphenicols	Macrolides	Amoxicillin/clavulanic acid						2		2				2
4	β_lactams	Amphenicols	Macrolides	Trimethoprim/Sulfamethoxazole					1	11	3	15		1	1	16
4	β_lactams	Amphenicols	Quinolones	Trimethoprim/Sulfamethoxazole					1			1				1
4	β_lactams	Amphenicols	Quinolones	Macrolides						1		1				1
4	β_lactams	Amphenicols	Lincosamides	Macrolides					2	9	1	12				12
4	Aminoglycosides	β_lactams	Trimethoprim/Sulfamethoxazole	Amoxicillin/clavulanic acid						1		1				1
4	Aminoglycosides	β_lactams	Quinolones	Amoxicillin/clavulanic acid						1		1	1		1	2
4	Aminoglycosides	β_lactams	Lincosamides	Macrolides						1		1				1
4	Aminoglycosides	β_lactams	Lincosamides	Quinolones						1		1				1
5	β_lactams	Quinolones	Macrolides	Trimethoprim/Sulfamethoxazole	Amoxicillin/clavulanic acid					3		3				3
5	β_lactams	Lincosamides	Quinolones	Macrolides	Amoxicillin/clavulanic acid				1	1		2				2
5	β_lactams	Lincosamides	Quinolones	Macrolides	Trimethoprim/Sulfamethoxazole				2	8		10				10
5	β_lactams	Amphenicols	Macrolides	Trimethoprim/Sulfamethoxazole	Amoxicillin/clavulanic acid					2		2				2
5	β_lactams	Amphenicols	Quinolones	Macrolides	Trimethoprim/Sulfamethoxazole					2	1	3				3
5	β_lactams	Amphenicols	Lincosamides	Macrolides	Amoxicillin/clavulanic acid					1	1	2				2
5	β_lactams	Amphenicols	Lincosamides	Macrolides	Trimethoprim/Sulfamethoxazole				6	25	1	32				32
5	β_lactams	Amphenicols	Lincosamides	Quinolones	Macrolides						1	1				1
5	Aminoglycosides	Lincosamides	Quinolones	Macrolides	Trimethoprim/Sulfamethoxazole					1		1				1
5	Aminoglycosides	β_lactams	Macrolides	Trimethoprim/Sulfamethoxazole	Amoxicillin/clavulanic acid				1			1				1
5	Aminoglycosides	β_lactams	Quinolones	Trimethoprim/Sulfamethoxazole	Amoxicillin/clavulanic acid				1			1				1
5	Aminoglycosides	β_lactams	Quinolones	Macrolides	Trimethoprim/Sulfamethoxazole					1		1				1
5	Aminoglycosides	β_lactams	Lincosamides	Macrolides	Trimethoprim/Sulfamethoxazole				2			2				2
5	Aminoglycosides	β_lactams	Amphenicols	Macrolides	Trimethoprim/Sulfamethoxazole					1		1				1
5	Aminoglycosides	β_lactams	Amphenicols	Lincosamides	Macrolides					6		6				6
6	β_lactams	Lincosamides	Quinolones	Macrolides	Trimethoprim/Sulfamethoxazole	Amoxicillin/clavulanic acid				7	3	10				10
6	β_lactams	Amphenicols	Lincosamides	Macrolides	Trimethoprim/Sulfamethoxazole	Amoxicillin/clavulanic acid			1	3	1	5	1		1	6
6	β_lactams	Amphenicols	Lincosamides	Quinolones	Macrolides	Amoxicillin/clavulanic acid				1		1		1	1	2
6	β_lactams	Amphenicols	Lincosamides	Quinolones	Macrolides	Trimethoprim/Sulfamethoxazole			6	14	6	26				26
6	Aminoglycosides	β_lactams	Quinolones	Macrolides	Trimethoprim/Sulfamethoxazole	Amoxicillin/clavulanic acid				1		1				1
6	Aminoglycosides	β_lactams	Lincosamides	Macrolides	Trimethoprim/Sulfamethoxazole	Amoxicillin/clavulanic acid							1		1	1
6	Aminoglycosides	β_lactams	Lincosamides	Quinolones	Macrolides	Trimethoprim/Sulfamethoxazole				3	1	4				4
6	Aminoglycosides	β_lactams	Amphenicols	Quinolones	Macrolides	Trimethoprim/Sulfamethoxazole					3	3				3
6	Aminoglycosides	β_lactams	Amphenicols	Lincosamides	Macrolides	Amoxicillin/clavulanic acid				1	2	3	1		1	4
6	Aminoglycosides	β_lactams	Amphenicols	Lincosamides	Macrolides	Trimethoprim/Sulfamethoxazole			1	17	1	19				19
6	Aminoglycosides	β_lactams	Amphenicols	Lincosamides	Quinolones	Macrolides				1	1	2				2
7	β_lactams	Amphenicols	Lincosamides	Quinolones	Macrolides	Trimethoprim/Sulfamethoxazole	Amoxicillin/clavulanic acid		4	4	1	9		1	1	10
7	Aminoglycosides	β_lactams	Lincosamides	Quinolones	Macrolides	Trimethoprim/Sulfamethoxazole	Amoxicillin/clavulanic acid			7	2	9				9
7	Aminoglycosides	β_lactams	Amphenicols	Quinolones	Macrolides	Trimethoprim/Sulfamethoxazole	Amoxicillin/clavulanic acid			1		1				1
7	Aminoglycosides	β_lactams	Amphenicols	Lincosamides	Macrolides	Trimethoprim/Sulfamethoxazole	Amoxicillin/clavulanic acid			4		4				4
7	Aminoglycosides	β_lactams	Amphenicols	Lincosamides	Quinolones	Macrolides	Amoxicillin/clavulanic acid			1	1	2				2
7	Aminoglycosides	β_lactams	Amphenicols	Lincosamides	Quinolones	Macrolides	Trimethoprim/Sulfamethoxazole		1	4		5				5
8	Aminoglycosides	β_lactams	Amphenicols	Lincosamides	Quinolones	Macrolides	Trimethoprim/Sulfamethoxazole	Amoxicillin/clavulanic acid	2	1		3	1		1	4*
				No. of MDR isolates					35	176	36	247	7	8	15	262
				No. of tested isolates					43	277	60	380	53	51	104	484

**S. pseudintermedius*
