# Supplementary material for: Antimicrobial resistance profiles of Staphylococcus spp. and Escherichia coli isolated from dogs and cats in Seoul, South Korea during 2021–2023
Source: Front Vet Sci. 2025 Aug 7;12:1563780. doi: 10.3389/fvets.2025.1563780 (PMC12367511; doi:10.3389/fvets.2025.1563780)
Supplement: Supplementary file 5 [file Table_5.pdf]

Supplementary Table 5. Distribution of MICs (μg/mL) in *E.coli* tested in this study (n=158).

Host	Antibiotics	MIC (μg/mℓ)*										Total
		≤0.12	0.25	0.5	1	2	4	8	16	32	≥64	
Dog	Amikacin					22	102	4				128
	Gentamicin		2	42	58	3			23			128
	Ampicillin				4	22	26	2	59	15		128
	Imipenem		20		107	1						128
	Cefovecin		2	46	37	2		10	31			128
	Cefpodoxime		8	1	74	4	1	9	31			128
	Cefalexin					2	24	48	11	33	10	128
	Chloramphenicol					2	19	68	21	1	17	128
	Doxycycline			2	29	45	15	13	24			128
	Tetracycline				12	3	72	4	6	31		128
	Enrofloxacin	52	7	15	8	2	13	31				128
	Marbofloxacin	46	8	22	10		12	30				128
	Pradofloxacin	5	74	4	3	1	41					128
	Trimethoprim/Sulphamethoxazole			79	17	1	1	23	7			128
	Amoxicillin/ Clavulanic Acid				2	8	49	38	3	28		128
Cat	Amikacin					8	21		1			30
	Gentamicin			13	12	1		4	4			30
	Ampicillin					9	3	1	13	4		30
	Imipenem		9		21							30
	Cefovecin		2	13	2	2		2	9			30
	Cefpodoxime		5	1	13			2	9			30
	Cefalexin						10	6	2	10	2	30
	Chloramphenicol					2	11	9	4		4	30
	Doxycycline			2	13	7	1	4	3			30
	Tetracycline				9		13			8		30
	Enrofloxacin	15	1	2	4		2	6				30
	Marbofloxacin	9	1	11	2	2		5				30
	Pradofloxacin	7	14		3		6					30
	Trimethoprim/Sulphamethoxazole			16	8			5	1			30
	Amoxicillin/ Clavulanic Acid					6	10	12		2		30

*White field indicates MIC ranges tested by either the Sensititre MIC system or the VITEK® 2 Compact system. Gray areas represent concentrations not tested by either method. MIC results for isolates collected in 2021 and 2022 were interpreted according to the guidelines of the Korean Animal and Plant Quarantine Agency, based on Clinical and Laboratory Standards Institute (CLSI) documents VET01S and M100 breakpoints. For isolates collected in 2023, antimicrobial susceptibility testing was performed using the VITEK® 2 Compact system, and interpretations were based on the "Result – Expertised" output generated by the system's Advanced Expert System. For the combinations, Trimethoprim/Sulphamethoxazole and Amoxicillin/Clavulanic Acid, the concentrations indicated are those of trimethoprim and Amoxicillin, respectively.
