# Supplementary material for: Antimicrobial resistance profiles of Staphylococcus spp. and Escherichia coli isolated from dogs and cats in Seoul, South Korea during 2021–2023
Source: Front Vet Sci. 2025 Aug 7;12:1563780. doi: 10.3389/fvets.2025.1563780 (PMC12367511; doi:10.3389/fvets.2025.1563780)
Supplement: Supplementary file 6 [file Table_6.pdf]

Supplementary table 6. Multi-drug resistance of *E. coli* isolated from dogs and cats in this study (*n* =158).

No. of antibiotics	Antibiotic resistance patterns						Dogs			Cats	Total
							Urine	Diarrhea	Total	Diarrhea	
0	None						20	5	25	10	35
1	Quinolones						2		2		2
1	β_lactams						4	23	27	6	33
1	Aminoglycosides						1		1		1
2	Tetracyclines	Trimethoprim/Sulfamethoxazole					2	1	3		3
2	β_lactams	Amoxicillin/clavulanic acid					5		5	2	7
2	β_lactams	Trimethoprim/Sulfamethoxazole					1	2	3	1	4
2	β_lactams	Quinolones					4	5	9	3	12
2	β_lactams	Tetracyclines						3	3		3
2	β_lactams	Amphenicols						1	1		1
2	Aminoglycosides	Tetracyclines						1	1		1
3	Tetracyclines	Quinolones	Trimethoprim/Sulfamethoxazole					1	1		1
3	β_lactams	Trimethoprim/Sulfamethoxazole	Amoxicillin/clavulanic acid				1		1		1
3	β_lactams	Quinolones	Amoxicillin/clavulanic acid				3		3		3
3	β_lactams	Tetracyclines	Trimethoprim/Sulfamethoxazole				3		3		3
3	β_lactams	Tetracyclines	Quinolones				2		2	1	3
3	β_lactams	Amphenicols	Quinolones				1		1		1
3	β_lactams	Amphenicols	Tetracyclines				2	1	3	2	5
3	Aminoglycosides	Tetracyclines	Trimethoprim/Sulfamethoxazole					1	1		1
3	Aminoglycosides	β_lactams	Trimethoprim/Sulfamethoxazole					1	1		1
3	Aminoglycosides	β_lactams	Quinolones				1	3	4		4
3	Aminoglycosides	β_lactams	Tetracyclines					1	1		1
4	β_lactams	Quinolones	Trimethoprim/Sulfamethoxazole	Amoxicillin/clavulanic acid			1		1		1
4	β_lactams	Tetracyclines	Quinolones	Amoxicillin/clavulanic acid			1		1		1
4	β_lactams	Tetracyclines	Quinolones	Trimethoprim/Sulfamethoxazole				1	1	1	2
4	β_lactams	Amphenicols	Quinolones	Amoxicillin/clavulanic acid			1		1		1
4	β_lactams	Amphenicols	Tetracyclines	Trimethoprim/Sulfamethoxazole				4	4		4
4	β_lactams	Amphenicols	Tetracyclines	Quinolones			3	1	4		4
4	Aminoglycosides	Tetracyclines	Quinolones	Trimethoprim/Sulfamethoxazole						1	1
4	Aminoglycosides	β_lactams	Quinolones	Amoxicillin/clavulanic acid			1		1		1
4	Aminoglycosides	β_lactams	Quinolones	Trimethoprim/Sulfamethoxazole			1		1		1
4	Aminoglycosides	β_lactams	Tetracyclines	Trimethoprim/Sulfamethoxazole						1	1
5	β_lactams	Amphenicols	Tetracyclines	Quinolones	Trimethoprim/Sulfamethoxazole			1	1		1
5	Aminoglycosides	β_lactams	Quinolones	Trimethoprim/Sulfamethoxazole	Amoxicillin/clavulanic acid		2		2		2
5	Aminoglycosides	β_lactams	Tetracyclines	Quinolones	Amoxicillin/clavulanic acid		1		1		1
5	Aminoglycosides	β_lactams	Tetracyclines	Quinolones	Trimethoprim/Sulfamethoxazole		1	1	2		2
5	Aminoglycosides	β_lactams	Amphenicols	Tetracyclines	Quinolones			1	1		1
6	Aminoglycosides	β_lactams	Amphenicols	Tetracyclines	Quinolones	Trimethoprim/Sulfamethoxazole	1	1	2	2	4
6	Aminoglycosides	β_lactams	Tetracyclines	Quinolones	Trimethoprim/Sulfamethoxazole	Amoxicillin/clavulanic acid	4		4		4
			No. of MDR isolates				30	18	48	8	56
			No. of tested isolates				69	59	128	30	158
